# Erythropoiesis-stimulating agents in oncology: a study-level meta-analysis of survival and other safety outcomes

**DOI:** 10.1038/sj.bjc.6605498

**Published:** 2010-01-05

**Authors:** J Glaspy, J Crawford, J Vansteenkiste, D Henry, S Rao, P Bowers, J A Berlin, D Tomita, K Bridges, H Ludwig

**Affiliations:** 1 Department of Medicine-Hematology and Oncology, David Geffen School of Medicine at University of California, Los Angeles, 100 UCLA Medical Plaza, Suite 550, Los Angeles, CA 90095-6996 USA; 2 Department of Medical Oncology, Duke University Medical Center, Box 3198, 25178 Morris Building, Durham, NC 27710 USA; 3 Respiratory Oncology Unit (Pulmonology), University Hospital Gasthuisberg, Herestraat 49, Leuven B-3000, Belgium; 4 Department of Medicine, Pennsylvania Hospital, 230 W Washington Square, Philadelphia, PA 19106 USA; 5 Johnson & Johnson Pharmaceutical Research and Development LLC, 1125 Trenton-Harbourton Road, PO Box 200, M/S K304, Titusville, New Jersey 08560 USA; 6 Amgen Inc., Thousand Oaks, CA 91320 USA; 7 First Department of Medicine, Center for Oncology and Haematology, Wilhelminenspital, Montleartstrasse 37, Vienna A-1171, Austria

**Keywords:** erythropoiesis-stimulating agent, anaemia, oncology, meta-analysis

## Abstract

BACKGROUND: Cancer patients often develop the potentially debilitating condition of anaemia. Numerous controlled studies indicate that erythropoiesis-stimulating agents (ESAs) can raise haemoglobin levels and reduce transfusion requirements in anaemic cancer patients receiving chemotherapy. To evaluate recent safety concerns regarding ESAs, we carried out a meta-analysis of controlled ESA oncology trials to examine whether ESA use affects survival, disease progression and risk of venous-thromboembolic events.

METHODS: This meta-analysis included studies from the 2006 Cochrane meta-analysis, studies published/updated since the 2006 Cochrane report, and unpublished trial data from Amgen and Centocor Ortho Biotech. The 60 studies analysed (15 323 patients) were conducted in the settings of chemotherapy/radiochemotherapy, radiotherapy only treatment or anaemia of cancer. Data were summarised using odds ratios (ORs) with 95% confidence intervals (CIs).

RESULTS: Results indicated that ESA use did not significantly affect mortality (60 studies: OR=1.06; 95% CI: 0.97–1.15) or disease progression (26 studies: OR=1.01; 95% CI: 0.90–1.14), but increased the risk for venous-thromoboembolic events (44 studies: OR=1.48; 95% CI: 1.28–1.72).

CONCLUSION: Though this meta-analysis showed no significant effect of ESAs on survival or disease progression, prospectively designed, future randomised clinical trials will further examine the safety and efficacy of ESAs when used according to the revised labelling information.

Cancer patients commonly develop anaemia due to either cancer itself or to treatments such as myelosuppressive chemotherapy (Groopman and Itri, 1999; Knight *et al*, 2004; Ludwig *et al*, 2004). During a large European survey of cancer patients with different tumour types and treatments, 67% of 13 628 patients were anaemic (defined as haemoglobin level <12 g dl^−1^ (7.5 mmol l^−1^)) at some time during the survey; anaemia was reported in 75% of those patients who received chemotherapy (Ludwig *et al*, 2004). Anaemia has been reported to be a potential risk factor for increased mortality, decreased efficacy of cancer treatments and longer hospitalisations (Schwartz, 2007; Spence, 2007). Anaemia can also increase fatigue, leading to reduced physical functioning and reduced quality of life (Curt *et al*, 2000). Although red blood cell (RBC) transfusions quickly correct anaemia, the effect is transient and transfusions carry inherent risks, including exposure to infectious agents (Barbara, 2004) and transfusion-related acute lung injury (Looney *et al*, 2004). A recent meta-analysis by the Cochrane Collaboration of randomised trials, prospective cohorts and retrospective surveys has also revealed that colorectal cancer patients receiving transfusions perioperatively have an increased risk for cancer recurrence (Amato and Pescatori, 2006).

Erythropoiesis-stimulating agents (ESAs) include recombinant erythropoietic glycoproteins developed as an anaemia-therapy alternative to RBC transfusions and their associated risks. ESAs approved in the United States of America (US) for treating chemotherapy-induced anaemia (CIA) in patients with non-myeloid malignancies include Epoetin alfa (Ortho, 2009) and darbepoetin alfa (Amgen, 2009). ESAs are also approved for this indication in the European Union (EU) (EMEA, 2008; eMC, 2008). Many clinical trials have demonstrated the efficacy and short-term safety of ESAs for raising and maintaining haemoglobin concentrations and for reducing RBC-transfusion rates (Glaspy *et al*, 1997; Demetri *et al*, 1998; Gabrilove *et al*, 2001; Littlewood *et al*, 2001; Vansteenkiste *et al*, 2002; Hedenus *et al*, 2003). Without using ESAs about 40–50% of CIA patients require transfusions (Littlewood *et al*, 2001; Vansteenkiste *et al*, 2002; Hedenus *et al*, 2003). That ESA therapy can reduce fatigue symptoms and measurably improve quality of life has been reported in several ESA trials (Vansteenkiste *et al*, 2002; Cella *et al*, 2003), meta-analyses of ESA studies (Ross *et al*, 2006; Minton *et al*, 2008) and in evidence-based ESA guidelines (Bokemeyer *et al*, 2007).

Though two trials suggested that ESA use might increase survival (enhanced treatment effects due to increased tumour-cell oxygenation was one postulated mechanism) (Littlewood *et al*, 2001; Vansteenkiste *et al*, 2002), several subsequent trials reported that targeting a haemoglobin level of ⩾12 g dl^−1^ in cancer patients receiving chemotherapy resulted in greater mortality in the ESA arm (Hedenus *et al*, 2003; Leyland-Jones *et al*, 2005; Amgen, 2007; ODAC, 2008). An ESA-associated increase in mortality and/or disease progression has also been reported in some controlled studies conducted in cancer settings off-label for ESA use, such as patients receiving radiotherapy only (Henke *et al*, 2003; Overgaard *et al*, 2007) or patients with anaemia not receiving radiotherapy or chemotherapy (i.e., anaemia of cancer (AoC)) (Wright *et al*, 2007; Smith *et al*, 2008). These safety signals have been the subject of three meetings (in 2004, 2007 and 2008) between the Oncologic Drugs Advisory Committee (ODAC) to the Food and Drug Administration (FDA) and the two US companies that distribute ESAs (Amgen and Centocor Ortho Biotech, Horsham, PA, USA, which is a subsidiary of Johnson & Johnson) (ODAC, 2008). Information on these safety signals has been added to the US and EU ESA-labelling information (EMEA, 2008; eMC, 2008; Amgen, 2009; Ortho, 2009). However, as an ESA-associated increase in mortality and disease progression has not been seen in a large number of other controlled ESA studies (Vansteenkiste *et al*, 2002; Blohmer *et al*, 2004; Machtay *et al*, 2007; Moebus *et al*, 2007; Pirker *et al*, 2008; Aapro *et al*, 2008a), those studies reporting adverse safety signals must be considered within the total body of evidence.

Meta-analysis is a well-established method to systematically evaluate available evidence, estimate an overall-treatment effect and explore sources of heterogeneity in reported findings (Egger *et al*, 1997). Four large meta-analyses of ESA trials in both approved and off-label oncology settings have recently been conducted (Bohlius *et al*, 2006; Ross *et al*, 2006; Seidenfeld *et al*, 2006; Bennett *et al*, 2008b). In the 2006 Cochrane Collaborative analysis (encompassing studies published or publicly available from 1985 to April 2005 that were conducted in the settings of chemotherapy only, radiotherapy with or without chemotherapy and AoC), 42 placebo- or standard-of-care-controlled ESA oncology trials were meta-analysed for survival (8167 patients) (Bohlius *et al*, 2005, 2006). The pooled results indicated a statistically non-significant numerical increase in mortality risk associated with ESA use (the odds ratio (OR) was 1.08 with a 95% confidence interval (CI) of 0.99–1.18 for ESA-treated patients *vs* control patients). Similar findings were reported in a meta-analysis by Ross *et al* (2006) and in the Seidenfeld *et al* (2006) meta-analysis from the US Agency for Healthcare Research and Quality (AHRQ) (Ross *et al*, 2006; Seidenfeld *et al*, 2006). Several smaller meta-analyses have also reported that ESA use has a non-significant effect on survival in cancer patients (Hedenus *et al*, 2005; Aapro *et al*, 2006, 2008b; Boogaerts *et al*, 2006). However, a recent large meta-analysis by Bennett *et al* (2008b) of 13 611 patients in 51 phase 3, controlled ESA oncology trials conducted in the chemotherapy, radiotherapy only and AoC settings indicated that mortality was significantly higher in the ESA group (Hazard ratio (HR)=1.10; 95% CI: 1.01–1.20; *P*=0.03) (Bennett *et al*, 2008b).

To comprehensively examine whether ESA use affects safety outcomes in cancer patients, we conducted a meta-analysis that included (1) studies included in the Cochrane 2006 meta-analysis, (2) controlled ESA studies in oncology published after April 2005 (the cut-off date for the Bohlius *et al* 2006 Cochrane review) up to March 2008 and (3) the most recent, updated ESA-trial data provided by Amgen and Centocor Ortho Biotech for unpublished and published trials (these data were not available for the recent Bennett *et al* (2008b) meta-analysis mentioned above). A total of 60 studies with survival data were identified, including all 51 studies analysed in Bennett *et al* (2008b). As the efficacy of ESAs for increasing haemoglobin levels and reducing transfusion requirements is well-established (Bohlius *et al*, 2006), our meta-analyses focused on the effect of ESA use on survival, disease progression and risk of venous-thromboembolic events (VTEs).

## Patients and methods

### Search strategy

The 2006 Cochrane Collaborative report (specifically Analysis 05.05) (Bohlius *et al*, 2006) was used as the basis for the present, updated meta-analysis. To identify studies published since the 2006 Cochrane report (i.e., from April 2005 to March 2008), we carried out a literature search of the BIOSIS Previews, Current Contents, EMBASE and Ovid MEDLINE databases using the following Boolean search string – ‘erythropoiesis stimulating proteins (including all synonymous terms)’ and ‘cancer (including ‘neoplasm’ and ‘malignancy’)’. A search was also carried out for relevant abstracts and associated poster presentations delivered from 1995 to 2007 at conferences for the American Society of Clinical Oncology (ASCO), American Society of Hematology (ASH), San Antonio Breast Cancer Symposium (SABCS) and the European Society for Medical Oncology (ESMO). Studies were selected if they were randomised, controlled trials of cancer patients treated with an ESA (Epoetin alfa, epoetin beta or darbepoetin alfa) plus transfusions compared with control patients who either received placebo or best standard of care for prophylaxis or treatment of anaemia (e.g., transfusions without ESAs). Studies had to report on death or percentage of death in each treatment group or to have collected mortality data that were available for analysis. Interim analyses of ESA trials were included, as well as ESA studies that examined iron use in cancer patients. One study (Blohmer *et al*, 2004) was rejected from the 2006 Cochrane analysis because oral iron was used in the ESA group and not the control group. This study was included in our analysis as it reported survival data, no evidence exists to suggest that oral iron supplementation affects mortality, and many ESA studies recommend iron supplementation to maintain adequate iron availability for erythropoiesis (Auerbach *et al*, 2004; Bastit *et al*, 2008).

We excluded those publications that were not written in English, that were editorials, letters, clinical guidelines or case studies, and that allowed ESAs to be administered to the control arm as part of standard medical care. If two publications existed describing the same study (e.g., an abstract and a manuscript), the most recent publication was used. For studies conducted by Amgen and Centocor Ortho Biotech, current data available from internal databases for those randomised, controlled ESA trials that met the search criteria used in the literature search were included in the analyses. Database results did not always match previously published results because longer-term follow-up data became available or because interim data were updated with either more recent interim data or the final data. In addition, death, disease progression and incidence of VTEs were frequently described as safety endpoints in the published literature and therefore, published studies usually analysed patients by treatment received. In this study-level meta-analysis, analyses used an intention-to-treat or modified-intention-to-treat approach (patients who received study drug were analysed). If data were not available by randomised-treatment assignment, only then were the published results used. Data for epoetin beta studies were collected from publications only and were not supplemented from internal databases at Hoffmann-La Roche (Basel, Switzerland).

Three modifications were made to the 2006 Cochrane analytical approach (Bohlius *et al*, 2006). First, our analysis collapsed chemotherapy groups used in the 2006 Cochrane meta-analysis to a single group, as ESA-labelling information does not differentiate between chemotherapy regimens with and without platinum and because the 2006 Cochrane mortality results were not significantly different between chemotherapy groups. Second, trials in which a majority of patients received chemotherapy (with or without radiotherapy) were classified as chemotherapy studies; in the 2006 Cochrane analysis, studies in which patients received radiotherapy and chemotherapy were classified as radiotherapy studies. Of note, one study labelled as a radiotherapy study in the 2006 Cochrane analysis (Wright *et al*, 2007) excluded patients who were to receive curative radiation. This study was classified as an AoC study in the present meta-analysis. Third, unlike the Cochrane analysis, we excluded studies in patients with myelodysplastic syndromes (MDS), as ESAs are not approved for use in MDS, which is a separate disease from cancer. One study included in the Cochrane 2006 report as an AoC study (Thompson *et al*, 2000) was excluded from the present meta-analysis because it was an MDS study.

Studies identified from the above literature search that also reported data on the relationship between ESA use and disease progression or VTE risk were meta-analysed to examine these relationships. Additional literature searches did not identify any additional controlled, ESA oncology studies reporting data on ESA use and disease progression or VTE risk.

### Statistical analyses

Data were summarised using ORs generated using the Comprehensive Meta-Analysis (V2) software. In contrast, the 2006 Cochrane Collaboration approach (Bohlius *et al*, 2006) reported either an OR or HR. As the method by which an OR is calculated provides a point estimate farther from unity (i.e., an estimate further from the null) than that provided by an HR, an OR is a more conservative estimate and may be more likely to detect a safety signal. Data are presented as a forest plot of all studies with an estimated OR and 95% CI. A random-effects model was used because it assumes treatment effects are not identical in all studies. Some analyses also used a fixed-effects model, which assumes that the treatment effect is the same in each study and that differences in results are due only to chance. Heterogeneity among studies was quantified using the *I*^2^ inconsistency statistic, which is a measure of inter-study variability as a proportion of total variability. When inter-study variability is low, the fixed-effects and random-effects models will yield very similar results.

For the present meta-analyses, both 1-year and long-term follow-up survival data were available from the Breast Cancer Erythropoietin Survival Trial (BEST study) (Leyland-Jones *et al*, 2005; Centocor Ortho Biotech, data on file). The 1-year BEST data were used in the figures. For completeness, results using the BEST long-term follow-up data are also provided within the text. The long-term data from BEST were collected after the end of the 1-year randomised period. For chemotherapy studies, an influence plot was generated that shows the estimated OR for mortality if an individual chemotherapy study (such as BEST) was excluded from the analysis.

As we did not have patient-level data for many of the studies included in this study-level meta-analysis, an overall-mortality HR was not calculated. However, sensitivity analyses were carried out on 20 chemotherapy studies with long-term follow-up (>6 months) to address concerns regarding the use of OR as a point estimate in a meta-analysis. Patient-level data from 16 of the 20 studies were obtained from Amgen and Centocor Ortho Biotech. In all, 4 of the 20 chemotherapy studies could not be included in the patient-level analysis as primary data were not available (Osterborg *et al*, 2005; Engert, 2007; Strauss *et al*, 2008; Aapro *et al*, 2008a). However, the published manuscripts for Aapro *et al* (2008a) and Osterborg *et al* (2005) provided HRs for survival. Thus, sensitivity analyses were also carried using the published HRs for these two studies. Data were combined and HRs for mortality were calculated for each study and across all studies combined (with study as a stratification factor) using an unadjusted Cox's proportional hazards model.

## Results

### Study selection

The literature search for controlled, ESA oncology trials that reported mortality or survival data identified 60 studies. In all, 19 were additional ESA studies that were added to 41 studies identified from the 2006 Cochrane meta-analysis of survival in ESA oncology trials (Bohlius *et al*, 2006) (Table 1). Of note, of the 42 studies included in the 2006 Cochrane survival analysis, 1 study was excluded from the present analysis as it was conducted in MDS patients (Thompson *et al*, 2000). Of the 19 additional studies, 12 were in the chemotherapy setting, 1 was in the radiotherapy alone setting and 6 were in AoC; for some of the 19 studies, updated, unpublished data from Amgen and Centocor Ortho Biotech were included in the present meta-analysis. Of the 41 studies selected from the Cochrane 2006 report, 14 were identified as having updated data available (e.g., results previously published in abstracts were now available as final publications or updated data were available from Amgen or Centocor Ortho Biotech) (Table 1). In this literature review, the 60 identified studies were conducted in patients with solid tumours and/or haematological malignancies who received an ESA (Epoetin alfa, epoetin beta or darbepoetin alfa) or control treatment (placebo or best standard of care). Only one study was conducted in paediatric patients (Razzouk *et al*, 2006). Of the 60 studies, 26 reported disease progression outcomes and 44 reported on the incidence of VTEs.

### Mortality

**Figure 1:**
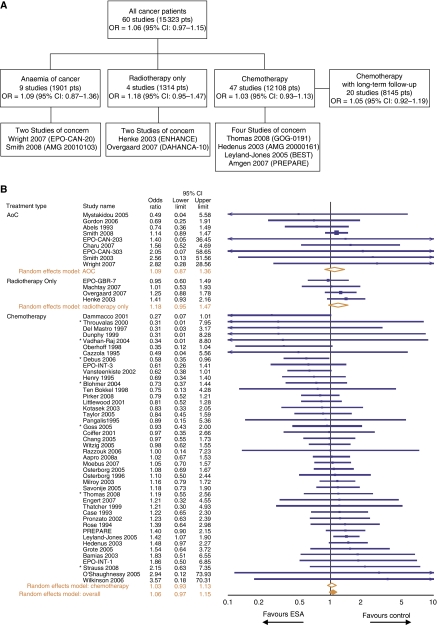

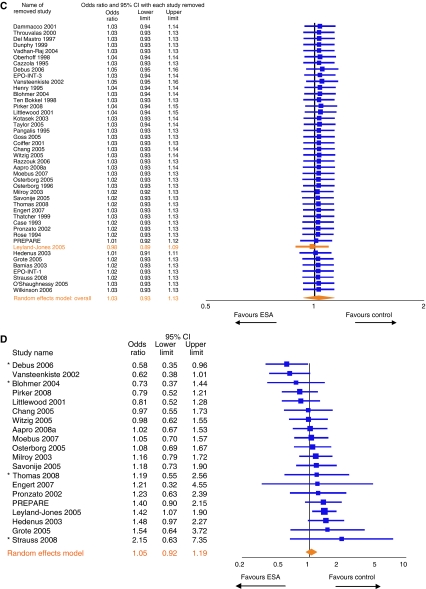
Effect of erythropoiesis-stimulating agent (ESA) use on mortality. (**A**) Flow diagram of studies analysed. The odds ratio (OR) (95% confidence interval (CI)) for the mortality of all 60 studies and for each cancer setting is given. Pts indicates patients. (**B**) Meta-analysis of mortality using 60 controlled studies carried out in the settings of chemotherapy, anaemia of cancer (AoC) or radiotherapy only. This analysis included 15 323 patients, with 8343 ESA-treated patients and 6980 control patients. An asterisk denotes radiochemotherapy studies. Three chemotherapy studies are not listed, as they reported no deaths in the ESA or control arms. (**C**) Influence of each chemotherapy study on the mortality OR for all chemotherapy studies. This analysis calculated the mortality OR for the chemotherapy studies combined after excluding each listed chemotherapy study. Of the 47 chemotherapy studies identified, 3 were not listed in the plot as they reported no deaths in the ESA or control arms. (**D**) Study-level meta-analyses of mortality in 20 chemotherapy studies with long-term follow-up. This analysis included 8145 patients, with 4183 ESA-treated patients and 3962 control patients. An asterisk denotes radiochemotherapy studies.

Mortality was assessed in 60 randomised, controlled trials (41 from the 2006 Cochrane analysis plus 19 additional studies) involving 15 323 patients (Figure 1). A total of 9 studies were in the AoC setting, 4 in the radiotherapy only setting and 47 in the chemotherapy setting. Of note, 3 of the 47 chemotherapy studies reported no deaths in either treatment arm (Cascinu *et al*, 1994; Kurz *et al*, 1997; Hedenus *et al*, 2002) and, thus, only 44 chemotherapy studies are listed in the plot shown in Figure 1B. Long-term follow-up (>6 months after the randomised study period) was reported in 26 studies (20 were chemotherapy studies), whereas the remaining 34 studies evaluated deaths on study.

Meta-analysis of all 60 studies based on a random-effects model yielded an OR for mortality of 1.06 (95% CI: 0.97–1.15; results were the same when a fixed-effects model was used) (Figure 1A and B; Table 2). There was little heterogeneity among the trials (*I*^2^=0%). This analysis included the 1-year survival data from the BEST study, which indicated higher mortality with ESA use in breast cancer patients (Leyland-Jones *et al*, 2005). However, this mortality signal has not been observed in long-term follow-up BEST data (ODAC, 2008). If long-term follow-up BEST data (i.e., survival data collected beyond the 1-year treatment period) were included (Leyland-Jones *et al*, 2005; ODAC, 2008), the mortality OR was 1.02 (95% CI: 0.94–1.11; *I*^2^=0%) using either the random-effects or fixed-effects model.

Meta-analysis of the 47 chemotherapy studies (12 108 patients) with either the random- or fixed-effects model resulted in an OR for mortality of 1.03 (95% CI: 0.93–1.13; I^2^=1.2%) (Figure 1A and B; Table 2). The BEST study provided the greatest weight (11.4%) in this analysis. The influence plot (Figure 1C) shows the effect of removing the BEST study (with 1-year data) from the meta-analysis of the chemotherapy studies; only removal of the BEST study shifted the mortality OR to <1. Meta-analysis of the 47 chemotherapy studies with the long-term follow-up BEST data resulted in a mortality OR of 0.98 (95% CI: 0.89–1.08) using either a random- or fixed-effects model. Meta-analyses of mortality in the off-label uses of AoC (9 studies; 1901 patients) and radiotherapy only (4 studies; 1314 patients) yielded an OR higher than that observed for the chemotherapy studies (Figure 1A and B; Table 2).

Studies in the chemotherapy setting that reported a mean-baseline haemoglobin concentration were analysed stratified by the baseline haemoglobin concentration at study entry. This analysis (Table 2) indicated a non-significant effect of ESAs on mortality in studies with a reported mean-baseline haemoglobin concentration of <10 g dl^−1^ or 10–12 g dl^−1^.

When mortality in the 20 chemotherapy studies with long-term follow-up data (8145 patients) was analysed, the mortality OR was 1.05 (95% CI: 0.92–1.19; *I*^2^=25.1%) using a random-effects model (Figure 1A and D) and 1.06 (95% CI: 0.96–1.18) using a fixed-effects model. If BEST long-term follow-up data were included (Leyland-Jones *et al*, 2005; ODAC, 2008), the mortality OR was 1.00 (95% CI: 0.89–1.12; *I*^2^=10.4%) using a random-effects model or 1.00 (95% CI: 0.90–1.11) using a fixed-effects model. Sensitivity analyses of the 20 chemotherapy trials with long-term follow-up survival data examined if OR estimates are effective for examining survival in meta-analyses. Study-level ORs and both study-level and patient-level HRs were estimated for mortality. Primary patient-level data (obtained from Amgen and Centocor Ortho Biotech) and data from the published literature were used. Though there are small variations, the estimates summarizing the mortality results across studies appeared to be consistent in indicating a small and non-significant effect of ESA use on mortality in the chemotherapy setting (Table 3).

### Disease progression

**Figure 2:**
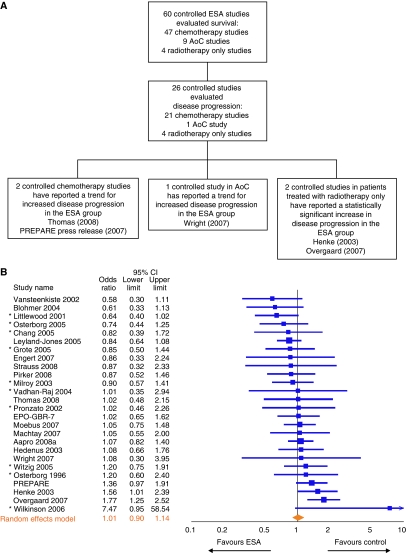
Effect of erythropoiesis-stimulating agent (ESA) use on disease progression. (**A**) Flow diagram of studies analysed. Of the 60 controlled ESA studies that measured survival, 26 also measured a disease-progression outcome. AoC indicates anaemia of cancer. (**B**) Study-level meta-analysis of disease progression-related endpoints in 26 controlled studies carried out in the settings of chemotherapy, AoC or radiotherapy only. This analysis included 9646 patients, with 4905 ESA-treated patients and 4721 control patients. An asterisk denotes studies where disease progression was evaluated only as part of tumour assessment.

Of the 60 controlled ESA trials that measured survival, 26 included a measure of disease progression (21 chemotherapy studies, 1 AoC study and 4 radiotherapy only studies) (Figure 2A). The disease-progression outcomes used (e.g., progression-free survival, relapse-free survival, local-regional relapse, tumour response or disease progression) and the quality of the tumour assessments employed varied. Only one study (AMG 20010145) (Pirker *et al*, 2008) rigorously assessed tumour progression radiographically using blinded centralised review. This small-cell lung cancer study in which all patients received the same chemotherapy regimen, reported that ESAs had no significant effect on disease progression (Pirker *et al*, 2008). In the remaining 25 studies, tumour progression was evaluated by the investigator (23 studies), by histopathology of tumour response (Amgen, 2007) or by radiographic assessment without central review (Grote *et al*, 2005).

To examine the effect of ESA use on disease progression in these 26 studies (9646 patients) and to standardise the variable outcome measures used to evaluate disease progression, ORs (ESA *vs* control) were calculated for each study (Figure 2B). Only the random-effects model was used since the many different disease progression estimates used in these studies would be expected to contribute to variability in the results. In the present meta-analysis, results indicated that ESA use was associated with a non-significant effect on disease progression (overall OR=1.01; 95% CI: 0.90–1.14; *I*^2^=29.4%) (Figure 2B). When the 21 chemotherapy studies (7908 patients with 4053 ESA-treated patients and 3855 control patients) that evaluated disease progression were meta-analysed, the result also indicated a non-significant effect of ESA use on disease progression (OR=0.95; 95% CI: 0.85–1.06; *I*^2^=2.3%).

### VTE risk

**Figure 3:**
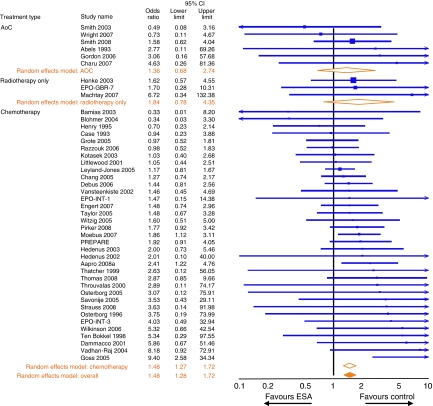
Study-level meta-analyses of venous-thromboembolic event (VTE) risk in 44 controlled studies carried out in the settings of chemotherapy, anaemia of cancer (AoC) or radiotherapy only. This analysis included 13 196 patients with 7237 ESA-treated patients and 5959 control patients.

An increased risk for VTEs with ESA use has been observed in individual, randomised controlled ESA studies and is consistently reported in the meta-analyses of ESA oncology trials (Table 4). The current meta-analysis examined 44 studies (13 196 patients) that reported the incidence of VTEs in the settings of chemotherapy (35 studies), AoC (6 studies) and radiotherapy only (3 studies). Results revealed that the risk of VTEs was increased in ESA-treated patients (OR=1.48; 95% CI: 1.28–1.72; *I*^2^=0% results were the same using either a random- or fixed-effects model) (Figure 3; Table 4). A similar result was seen when the 35 chemotherapy studies were analysed (OR=1.48; 95% CI: 1.27–1.72; *I*^2^=0%). A meta-analysis for VTE risk carried out in the 18 chemotherapy studies with long-term follow-up (6498 patients with 3859 ESA-treated patients and 2639 control patients; these studies were analysed as their reported VTE definitions were the most consistent) also indicated an increased VTE risk with ESA use (OR=1.47; 95% CI: 1.24–1.74; *I*^2^=0% results were the same using either a random- or fixed-effects model).

## Discussion

The present meta-analysis of studies in the chemotherapy, radiotherapy and AoC oncology settings indicates that ESA use is associated with a significant increase in VTE risk (44 studies examined), but does not demonstrate a significant effect on mortality (60 studies examined) or on disease progression (26 studies examined). However, the US and EU ESA-labelling information describes how increased mortality and/or disease progression has been observed in 8 individual studies included in this meta-analysis: 4 in the chemotherapy setting (Hedenus *et al*, 2003; Leyland-Jones *et al*, 2005; Amgen, 2007; Thomas *et al*, 2008), 2 in the radiotherapy setting (Henke *et al*, 2003; Overgaard *et al*, 2007) and 2 in the AoC setting (Wright *et al*, 2007; Smith *et al*, 2008). The current EU and US ESA-labelling information recommends that ESAs not be used in AoC or radiotherapy alone settings and that ESA therapy be initiated in patients with a baseline haemoglobin ⩽10 g dl^−1^ (EU) or <10 g dl^−1^ (US).

Three other large study-level meta-analyses have also shown that ESA use has a non-significant effect on survival in oncology patients (Bohlius *et al*, 2006; Ross *et al*, 2006; Seidenfeld *et al*, 2006) (Table 2). In contrast, the recent meta-analysis by Bennett *et al* (2008b) indicated that ESA-treated patients had significantly increased mortality (Table 2). The discrepancy between this finding and the findings from other meta-analyses, including this study, may be because of differences in the data or studies included for analysis (Bohlius *et al*, 2008). The present meta-analysis included all 51 studies used in the Bennett *et al* (2008b) study. However, a few studies included in the current analysis were not included in the Bennett *et al* (2008b) meta-analysis (Bennett *et al*, 2008b) or in a subsequent analysis performed by Bennett *et al* (2008a) that was described in a letter to the editor. In addition, this study included updated data (available either in recent publications or in unpublished study updates) not available to Bennett *et al* (2008b).

Of further interest, two meta-analyses examining the effect of ESA use on mortality in cancer patients were published after the present meta-analysis was completed. One was a patient-level meta-analysis carried out by the Cochrane EPO Individual Patient Data (IPD) Meta-analysis Collaborative group (data were supplied by Amgen, Centocor Ortho Biotech, Hoffman-La Roche and several large investigator-initiated studies) (Bohlius *et al*, 2009) and the other was a study-level meta-analysis carried out by the Canadian Agency for Drugs and Technologies in Health (Tonelli *et al*, 2009). In the Bohlius *et al* (2009) study, an analysis of chemotherapy studies (38 studies with 10 441 patients) indicated that the overall-survival HR was 1.04 (95% CI: 0.97–1.11) and the HR for deaths on study was 1.10 (95% CI: 0.98–1.24) (Bohlius *et al*, 2009). In all oncology settings (53 trials with 13 933 patients), the HR for overall survival was 1.06 (95% CI: 1.00–1.12) and the HR for on-study mortality was 1.17 (95% CI: 1.06–1.30). Though the overall-survival HR for all oncology settings is nearly identical to the mortality OR estimated in the current meta-analysis, the HR for on-study mortality reported by Bohlius *et al* (2009) for all oncology settings indicated an increased risk of death with ESA use. Differences between results reported by Bohlius *et al* (2009) and results described here may reflect slight differences in the methods and data used. The Bohlius *et al* (2009) study used a straight intention-to-treat approach, whereas the current meta-analysis used a modified intention-to-treat approach when data were available for such an approach (only patients who received at least one dose of study drug were analysed). In addition, although the Bohlius *et al* (2009) meta-analysis used patient-level data, the current meta-analysis only had access to published study-level data for those trials or investigator-sponsored studies sponsored by Hoffman-La Roche.

Similar to the current meta-analysis, the Tonelli *et al* (2009) meta-analysis indicated a non-significant effect of ESA use on mortality when 23 controlled trials (4273 patients) conducted in the chemotherapy setting were examined (relative risk=1.04; 95% CI: 0.86–1.26) (Tonelli *et al*, 2009). However, an analysis of 28 controlled ESA trials (6525 patients) in all oncology settings indicated that ESA use was associated with a higher relative risk for mortality (relative risk=1.15; 95% CI: 1.03–1.29) (Tonelli *et al*, 2009). Differences in the results reported by Tonelli *et al* (2009) and by the current meta-analysis may reflect differences in the studies analysed. Unlike the current meta-analysis, the Tonelli *et al* (2009) meta-analysis included publications written in a foreign language and studies conducted in a peri-surgical setting. In addition, the Tonelli *et al* (2009) meta-analysis did not include some key ESA trials (such as the BEST study by Leyland-Jones *et al*, 2005), as anaemia due to chemotherapy was not a study entry criterion.

Inconsistent results have also been reported across individual ESA studies with regard to when an ESA-associated mortality signal arises and in what tumour type it occurs. In chemotherapy studies with high haemoglobin targets (⩾12 g dl^−1^), decreases in survival have been observed both during the treatment period (e.g., in the first 4 months in the BEST study) (Leyland-Jones *et al*, 2005) and only after years of follow-up (ODAC, 2008; Hedenus *et al*, 2003 and Amgen, data on file). For every study listed in the US ESA-labelling information reporting an ESA-associated increase in mortality and/or disease progression in a specific tumour type (Amgen, 2009; Ortho, 2009), there is a study conducted in a similar tumour type that did not report these safety signals. For example, in cervical cancer, the GOG-0191 study (in 114 radiochemotherapy patients out of a planned 460) showed a non-significant trend of increased disease progression and increased mortality in ESA-treated patients at 3 years (Thomas *et al*, 2008). In contrast, a similar study in cervical cancer (250 radiochemotherapy patients) showed no difference in the overall survival between treatment arms after 4.8 years of follow-up (Blohmer *et al*, 2004). In breast cancer, an unplanned analysis of 3-year interim data from the Preoperative Epirubicin Paclitaxel Aranesp (PREPARE) study (733 chemotherapy patients) reported increased mortality and increased disease progression in ESA-treated patients (Amgen, 2007). However, final 5-year follow-up results from another breast cancer study (643 chemotherapy patients) showed no survival difference between treatment groups (Moebus *et al*, 2007). Inconsistent safety signals have also been observed in other cancer types, most notably haematological malignancies (Osterborg *et al*, 1996, 2005; Hedenus *et al*, 2003). The current EU and US ESA-labelling information contain no restrictions on ESA use by tumour type (EMEA, 2008; eMC, 2008; Amgen, 2009; Ortho, 2009).

The mechanism behind the ESA-associated increase in mortality seen in some studies is unclear. One hypothesised mechanism is that ESAs could stimulate disease progression by activating erythropoietin receptors (EpoRs) that might be present on tumour cells and/or tumour-associated vasculature. Several immunohistochemistry studies using polyvalent rabbit anti-sera raised against EpoR (i.e., polyclonal antibodies for EpoR) have reported EpoR expression in some primary human tumours and endothelial cells (reviewed in Osterborg *et al*, 2007; Sinclair *et al*, 2007). Several studies have also reported that recombinant human erythropoietin might have a proliferative or survival-promoting effect on tumours (Takeshita *et al*, 2000; Westenfelder and Baranowski, 2000; Acs *et al*, 2001; Lai *et al*, 2005; Feldman *et al*, 2006). However, the majority of pre-clinical studies have failed to detect such effects when human tumour cell lines (in cell culture or in rodent tumour models) are exposed to ESAs, even at supra-pharmacological ESA concentrations (Osterborg *et al*, 2007; Sinclair *et al*, 2007). Furthermore, several investigators have independently demonstrated that commercially available EpoR antibodies lack specificity and provide false-positive results with immunohistochemistry (Elliott *et al*, 2006; Brown *et al*, 2007; Sinclair *et al*, 2007; Laugsch *et al*, 2008). Recent studies have also found that tumour cell lines and tumour biopsies do not contain increased levels of EpoR mRNA transcripts compared with normal tissue controls and that the EpoR gene is not amplified in tumour cells (Sinclair *et al*, 2008). Overall, the weight of preclinical evidence does not support a role for EpoR in tumour growth. A recent workshop at the National Cancer Institute (December 2007) concluded that conflicting and confounding data exist regarding the expression and function of EpoR protein on tumour cells and the association of EpoR with disease progression. Additional research with more specific EpoR-detection agents is needed to examine questions regarding EpoR and disease progression.

Examining ESA use and disease progression is further complicated by the fact that disease progression has not been a primary or secondary endpoint in most oncology ESA studies. In addition, great variation exists in the progression endpoints measured (e.g., progression-free survival, tumour response, local-regional relapse, and so on) and in the quality, consistency and frequency of tumour assessments. Further, only those chemotherapy studies targeting high haemoglobin levels (⩾12 g dl^−1^) have reported increased mortality with ESA use. If an ESA-associated mortality risk is due to increased disease progression, then this mortality risk would be expected to be observed regardless of the target haemoglobin level. Additional controlled studies are needed that prespecify disease progression as a primary or secondary endpoint and that use consistent methods with central review to collect and report the disease-progression data.

Though an ESA-associated mortality signal in the chemotherapy setting is only seen in studies targeting a high haemoglobin level (⩾12 g dl^−1^), not all such chemotherapy studies have reported this signal (Pirker *et al*, 2008). Thus, the mechanism responsible for ESA-associated mortality is unlikely to be solely related to the high haemoglobin target. An exploratory analysis of six controlled darbepoetin alfa chemotherapy trials indicated that patients who responded poorly to ESA therapy (i.e., haemoglobin levels rose very little or not at all; *n*=1089) had a two-fold higher mortality rate relative to patients who responded well to ESA therapy (*n*=385) (Amgen, data on file). Though poor ESA responders appeared to drive the mortality signal in these chemotherapy studies, reasons for the increased mortality were unclear. This finding is notably confounded in that a poor response to ESA therapy may be a marker for those patients with a poorer prognosis (ODAC, 2008). The current ESA-labelling information recommends halting ESAs in cancer patients who have no haemoglobin response.

Poor ESA response as a driver for a mortality signal in studies with high haemoglobin target has also been observed in the nephrology setting. In both the Normal Hematocrit Cardiac Trial (NHCT) (Besarab *et al*, 1998) and the Correction of Hemoglobin in the Outcomes in Renal Insufficiency (CHOIR) trial (Singh *et al*, 2006), patients randomised to arms with higher haemoglobin targets had an elevated risk for a composite event of cardiovascular hospitalisation and death. However, those patients who achieved higher haemoglobin levels in both the lower haemoglobin-target arms and the higher haemoglobin-target arms had better outcomes. A re-analysis of data from the NHCT higher haemoglobin-target arm showed that patients with the lowest haemoglobin response had the highest mortality risk, which was independent of other mortality predictors (Kilpatrick *et al*, 2008).

The well-characterised risk of VTEs associated with ESA use has been posed as a possible mechanism for the ESA-associated mortality observed in some ESA studies. An increased VTE risk with ESA use in the oncology setting is documented in the scientific literature (Bohlius *et al*, 2006), in the US ESA-labelling information (Amgen, 2007; Ortho, 2009), and is also supported by the present meta-analysis. If poor ESA response drives the mortality signal and the mechanism for increased mortality is VTE incidence, the implication is that ESA exposure can increase VTE risk by a mechanism unrelated to haemoglobin rise, as poor responders show little or no increase in haemoglobin. An alternate mechanism could be an effect of ESAs on Virchow's triad of factors that affect the pathogenesis of thrombosis, including hypercoagulability, vessel-wall injury and venous stasis (Baron *et al*, 1998; Lee, 2002). Studies examining survival in ESA-treated cancer patients treated with prophylactic low-molecular-weight heparin (to decrease VTEs) (Geerts *et al*, 2004) would be enlightening.

Studies are needed to further explore the effect of ESA use on survival and disease progression, to definitively determine what can drive ESA-associated mortality, and to evaluate factors that may identify patients most at risk for increased mortality with ESA use. Important information will be provided from ongoing clinical trials such as the PREPARE trial and the EPO-ANE-3010 trial, which is an open-label, randomised and controlled trial in metastatic breast cancer patients measuring progression-free survival (primary endpoint) and overall survival (secondary endpoint) (Centocor Ortho Biotech, data on file). A large, placebo-controlled, non-inferiority study in non-small-cell lung cancer has been proposed in which randomisation will be stratified by prognostic factors (study 20070782; Amgen, data on file) and overall survival (primary endpoint) and progression-free survival (secondary endpoint) will be examined. Results from these and additional trials and from inclusive meta-analyses will provide important evidence to inform evidence-based decision making.

## Figures and Tables

**Table 1:** Characteristics of the 60 studies examined in the present meta-analysis

**Study publication**	**Study number or alias**	**Treatment setting**	**Tumour type**	**Number of patients analysed**
*Studies not present in Cochrane 2006*				
a Aapro *et al*, 2008a	BRAVE	Chemotherapy	Breast	463
a,b Blohmer *et al*, 2004	AGO/NOGG EPO-GER-8	Chemotherapy	Cervical	250
Charu *et al*, 2007	AMG 20000219	AoC	Non-myeloid	285
a,b Debus *et al*, 2006	EPO-GER-22	Chemotherapy	NSCLC	385
Engert, 2007	HD-15	Chemotherapy	Hodgkin's lymphoma	688
Gordon *et al*, 2006	AMG 20030204	AoC	Non-myeloid	218
a Milroy *et al*, 2003	EPO-INT-49	Chemotherapy	NSCLC	424
Moebus *et al*, 2007	EPO-GER-7	Chemotherapy	Breast	643
Mystakidou *et al*, 2005	N/A	AoC	Solid tumour	100
Overgaard *et al*, 2007	SE-2002-9001 (DAHANCA-10)	Radiotherapy	Head and neck	515
a Pirker *et al*, 2008	AMG 20010145	Chemotherapy	SCLC	596
Pronzato *et al*, 2002	EPO-INT-47	Chemotherapy	Breast	223
a Smith *et al*, 2008	AMG 20010103	AoC	Non-myeloid	985
b Strauss *et al*, 2008	MARCH	Chemotherapy	Cervical	74
Taylor *et al*, 2005	AMG 20030232	Chemotherapy	Non-myeloid	386
Wilkinson *et al*, 2006	EPO-INT-45	Chemotherapy	Ovarian	181
aUnpublished	PREPARE	Chemotherapy	Breast	733
Unpublished	EPO-CAN-203	AoC	N/A	17^c^
Unpublished	EPO-CAN-303	AoC	N/A	16^c^
				
*Studies included in Cochrane 2006 that had updates since Cochrane 2006*				
Chang *et al*, 2005	EPO-CAN-17	Chemotherapy	Breast	354
b Goss *et al*, 2005	EPO-CAN-15	Chemotherapy	SCLC	104
a Grote *et al*, 2005	N93-004	Chemotherapy	SCLC	224
a Hedenus *et al*, 2003	AMG 20000161	Chemotherapy	Haematological	344
a Leyland-Jones *et al*, 2005	EPO-INT-76 (BEST)	Chemotherapy	Breast	939
a Machtay *et al*, 2007	RTOG-99-03 PR99-03-046	Radiotherapy	Head and neck	148
Razzouk *et al*, 2006	CR002296	Chemotherapy	Solid or various haematological	224
Savonije *et al*, 2005	EPO-NED-17	Chemotherapy	Solid tumours	315
a,b Thomas *et al*, 2008	GOG-0191	Chemotherapy	Cervical	114
a Vansteenkiste *et al*, 2002	AMG 980297	Chemotherapy	SCLC and NSCLC	314
a Wright *et al*, 2007	EPO-CAN-20	AoC	NSCLC	70
Unpublished	EPO-INT-1	Chemotherapy	Ovarian	246
Unpublished	EPO-INT-3	Chemotherapy	Solid or various haematological	201
aUnpublished	EPO-GBR-7	Radiotherapy	Head and neck	300
				
*Studies included in Cochrane 2006 that have had no updates*				
Abels, 1993	N/A	AoC	Mixed	124
Bamias *et al*, 2003	N/A	Chemotherapy	Solid tumour	144
Cascinu *et al*, 1994	N/A	Chemotherapy	Mixed	100
Case *et al*, 1993	N/A	Chemotherapy	Mixed	157
Cazzola *et al*, 1995	MF4313	Chemotherapy	Multiple myeloma or non-Hodgkin's lymphoma	146
Coiffier *et al*, 2001	MF4421	Chemotherapy	Mixed	262
Dammacco *et al*, 2001	EPO-INT-2	Chemotherapy	Multiple myeloma	145
Del Mastro *et al*, 1997	N/A	Chemotherapy	Breast	62
Dunphy *et al*, 1999	N/A	Chemotherapy	Head and neck or lung carcinoma	30
Hedenus *et al*, 2002	AMG 990114	Chemotherapy	Lymphoproliferative	66
Henke *et al*, 2003	ENHANCE	Radiotherapy	Head and neck	351
Henry *et al*, 1995	N/A	Chemotherapy	Mixed	132
Kotasek *et al*, 2003	AMG 980291	Chemotherapy	Solid tumours	249
Kurz *et al*, 1997	N/A	Chemotherapy	Gynaecological	35
a Littlewood *et al*, 2001	EPO-INT-10	Chemotherapy	Solid or non-myeloid malignancy	375
Oberhoff *et al*, 1998	N/A	Chemotherapy	Solid tumours	218
O’Shaughnessy *et al*, 2005	PR00-27-005	Chemotherapy	Breast	100
Osterborg *et al*, 1996	MF4250	Chemotherapy	Haematological	144
a Osterborg *et al*, 2005	MF4467	Chemotherapy	Haematological	343
Pangalis *et al*, 1995	P-174	Chemotherapy	B-chronic lymphocytic leukaemia	45
Rose *et al*, 1994	J89-040	Chemotherapy	Chronic lymphocytic leukaemia	221
Smith *et al*, 2003	AMG 990111	AoC	Mixed	86 (Q3W/Q4W)
ten Bokkel Huinink *et al*, 1998	N/A	Chemotherapy	Ovarian	120
Thatcher *et al*, 1999	CC2574-P-169	Chemotherapy	SCLC	130
b Throuvalas *et al*, 2000	N/A	Chemotherapy	Cervix or bladder	55
b Vadhan-Raj *et al*, 2004	PR00-03-006	Chemotherapy	Gastric and rectal	60
Witzig *et al*, 2005	PR98-27-008	Chemotherapy	Mixed	344

AoC=anaemia of cancer; N/A=not available; NSCLC=non-small-cell lung cancer; SCLC=small-cell lung cancer; Q3W=every 3 weeks; Q4W=every 4 weeks.

a These studies have reported a mortality hazard ratio that is publicly available in a journal or abstract publication, the ODAC (2008) briefing book or in Hedenus *et al* (2005).

b Patients received chemotherapy and radiotherapy.

c The planned enrolment was 160 patients for EPO-CAN-203 and 540 patients for EPO-CAN 303. Both studies were stopped early because of poor accrual (Centocor Ortho Biotech, data on file).

**Table 2:** Risk of death in patients receiving ESAs compared with patients not receiving ESAs: results from five meta-analyses

	**Number of trials**	**Number of patients**	**Statistic**	**95% Confidence interval**
*Current meta-analysis, All studies*	60	15 323	1.06 OR	0.97–1.15
Chemotherapy studies	47	12 108	1.03 OR	0.93–1.13
Mean entry haemoglobin <10 g 100 ml^−1^	16	3265	0.99 OR	0.80–1.22
Mean entry haemoglobin 10–12 g 100 ml^−1^	13	3661	0.91 OR	0.77–1.08
Mean entry haemoglobin >12 g 100 ml^−1^	13	4522	1.13 OR	0.94–1.36
AoC studies	9	1901	1.09 OR	0.87–1.36
Radiotherapy studies	4	1314	1.18 OR	0.95–1.47
*Cochrane (Bohlius *et al*, 2006), All studies*	42	8167	1.08 OR	0.99–1.18
All studies, entry haemoglobin <10 g 100 ml^−1^	20	3765	1.01 OR	0.89–1.15
All studies, entry haemoglobin 10–<12 g 100 ml^−1^	8	1712	0.98 OR	0.82–1.16
All studies, entry haemoglobin >12 g 100 ml^−1^	7	1696	1.27 OR	1.05–1.54
Chemotherapy studies^a^	30	6282	1.02 OR	0.90–1.15
AoC studies	3	276	1.14 OR	0.56–2.31
Radiotherapy studies	8	1187	1.27 OR	1.05–1.55
*AHRQ (Seidenfeld *et al*, 2006), All studies*	39	7891	1.08 Peto OR	0.98–1.18
All studies, entry haemoglobin <10 g 100 ml^−1^	17	3489	1.01 Peto OR	0.89–1.15
All studies, entry haemoglobin 10–12 g 100 ml^−1^	8	1712	0.98 Peto OR	0.82–1.16
All studies, entry haemoglobin >12 g 100 ml^−1^	7	1696	1.27 Peto OR	1.05–1.54
*(Ross *et al*, 2006), All studies*	17	2895	1.14 OR	0.90–1.45
CIA studies (entry haemoglobin <11 g 100 ml^−1^)	11	2014	0.99 OR	0.72–1.36
Non-CIA studies	6	881	1.39 OR	0.96–2.00
*(Bennett *et al*, 2008b), All studies*	51	13 611	1.10 HR	1.01–1.20
Chemotherapy or radiotherapy studies	45	11 522	1.09 HR	0.99–1.19
AoC studies	6	1800	1.29 HR	1.00–1.67

All studies=all studies analysed from settings of chemotherapy, anaemia of cancer and radiotherapy only; AoC=anaemia of cancer; CIA=chemotherapy-induced anaemia; ESA=erythropoiesis-stimulating agent; HR=hazard ratio; OR=odds ratio.

a Estimated by Amgen based on original classification of studies from the Cochrane 2006 meta-analysis (Bohlius *et al*, 2006).

**Table 3:** Sensitivity analysis of odds ratio^a^ *vs* hazard ratio^b^ for estimating mortality in chemotherapy studies with long-term follow-up

**Number of studies analysed**	**Studies excluded**	**Summary measure**	**Point estimate**	**95% CI**
20	None	Study-level OR	1.05	0.92–1.19
18	Engert, 2007; Strauss *et al*, 2008	Study-level HR	1.04	0.95–1.14
16	Engert, 2007 Osterborg *et al*, 2005; Aapro *et al*, 2008a; Strauss *et al*, 2008	Study-level OR	1.04	0.89–1.21
16	Engert, 2007; Osterborg *et al*, 2005 Aapro *et al*, 2008a; Strauss *et al*, 2008	Study-level HR	1.04	0.94–1.15
16	Engert, 2007 Osterborg *et al*, 2005 Aapro *et al*, 2008a; Strauss *et al*, 2008	Patient-level HR	1.03	0.95–1.11

CI=confidence interval; HR=hazard ratio; OR=odds ratio.

a ORs were calculated for 20 studies with long-term follow-up data, for 16 studies where primary data were available and for 18 studies with either a reported or calculated HR. The OR was based only on the number of deaths.

b HRs were calculated for 16 studies where primary data were available. Both study-level and patient-level analyses are provided.

**Table 4:** Risk of venous thromboembolism for patients receiving ESAs compared with patients not receiving ESAs: results from five meta-analyses^a^

**Meta-analysis**	**Number of trials**	**Number of patients**	**Statistic for VTE risk**	**95% Confidence interval**
Present meta-analysis	44	13 196	1.48 OR	1.28–1.72
Bohlius *et al*, 2006	35	6769	1.67 RR	1.35–2.06
Seidenfeld *et al*, 2006	31	6412	1.68 RR	1.36–2.08
Ross *et al*, 2006	6	1463	1.41 OR	0.81–2.47
Bennett *et al*, 2008b	38	8172	1.57 RR	1.31–1.87

ESA=erythropoiesis-stimulating agent; OR=odds ratio; RR=relative risk; VTE=venous-thromboembolic event.

a Results from studies conducted in the chemotherapy, radiotherapy and anaemia of cancer (AoC) settings are included.
